# Innovative Postharvest Management for Hass Avocado at the Preclimacteric Stage: A Combined Technology with GABA and 1-MCP

**DOI:** 10.3390/foods13162485

**Published:** 2024-08-07

**Authors:** María Celeste Ruiz-Aracil, Juan Miguel Valverde, Mihaela Iasmina Madalina Ilea, Daniel Valero, Salvador Castillo, Fabián Guillén

**Affiliations:** Postharvest Research Group of Fruit and Vegetables, CIAGRO, University Miguel Hernández, Ctra. Beniel km. 3.2, 03312 Orihuela, Alicante, Spain; maria.ruiz61@goumh.umh.es (M.C.R.-A.); jm.valverde@umh.es (J.M.V.); mihaela.ilea@alu.umh.es (M.I.M.I.); daniel.valero@umh.es (D.V.); scastillo@umh.es (S.C.)

**Keywords:** *Persea americana* Mill, cold tolerance, shelf life, ripening, γ-aminobutyric acid, 1-methylcyclopropene

## Abstract

Avocado (*Persea americana* Mill.) is a subtropical climacteric fruit with a limited shelf life due to its high sensitivity to low temperatures. Chilling injury (CI) produced by cold storage displays symptoms in avocado fruit such as irregular ripening, darkening of the mesocarp, hardening of vascular strands, lipid oxidation with “off flavors”, and pitting and darkening of the skin, increasing weight loss. Accordingly, we studied the effect of γ-aminobutyric acid (GABA) and 1-methylcyclopropene (1-MCP) alone or in combination as postharvest treatments to maintain quality and to increase cold tolerance. Hass avocados were stored at 5 °C plus 5 days at room temperature. The results showed that the combined treatment improved fruit quality parameters as compared with control fruit and with those treated with only 1-MCP or GABA. The combined treatment delayed synergistically the postharvest ripening process. This delayed pattern was concomitant with a delayed ethylene pattern in GABA + 1-MCP or 1-MCP fruit batches. CI symptoms and electrolyte leakage were minimized in all GABA and 1-MCP fruit batches specifically in the combined treatment. For this reason, the synergistic effect of the combination of treatments may be recommended as an effective alternative strategy to prolong the postharvest quality of avocado during refrigerated storage.

## 1. Introduction

Avocado (*Persea americana* Mill.) is native to central and southern Mexico and is a fruit grown worldwide and with nutraceutical attributes, specifically the Hass variety [1]. This fruit is known for its nutritional content and human health benefits, which are primarily due to the source of fat-soluble nutrients or phytochemicals such as xanthophyll, carotenoids, phenols and phytosterols [2]. The consumption of Hass avocados has been linked to beneficial effects, including anticarcinogenic and antioxidant properties, as well as a reduced risk of cardiovascular diseases and obesity [2].

The avocado fruit is very susceptible to mechanical damage, particularly bruising and to chilling injury (CI) during cold storage, as well as physiological breakdown and microbial spoilage. For this reason, ensuring the highest fruit quality and preventing spoilage during transportation or commercial distribution is a significant challenge in avocado fruit due to their delicate nature [3]. Avocados have significant postharvest losses up to 43% according to Yahia et al. [4]. This places avocados among the fruits with some of the highest loss percentages. Recent research establishes that postharvest losses for fruits and vegetables worldwide range from 28 to 55%, depending on the plant species [5]. This highlights the urgent need for technological improvements. This is especially important for avocados, not just to help with distribution but also to enhance storability ensuring a longer shelf life and reducing waste after harvesting. In addition, avocado is a climacteric fruit, meaning that once harvested, it can continue to ripen due to its high respiration rate and abundant ethylene production [6]. Mishandling can greatly diminish the shelf life of avocados, leading to water loss, premature ripening, and structural decay. These issues have a direct impact on the economic viability of both producers and retailers. Several factors stimulate the avocado senescence, including the level of fruit ripening, metabolic and respiration rates, ethylene production, accumulation of carbon dioxide, and the heat generated during the climacteric process. These aspects collectively contribute to the degradation of avocado quality over time [7].

The initial postharvest strategies in the fruit supply chain involve selection and categorization processes aimed at minimizing postharvest losses. To ensure avocados can endure prolonged transportation periods, the level of ripeness often serves as a key classification criterion [8]. On the other hand, storage temperature is one of the most usual and effective technologies to delay fruit ripening, managing to reduce the climacteric increase in respiration, in addition to controlling internal CO_2_ outflow and external oxygen inflow [9]. However, exposure of avocados to suboptimal temperatures below their critical threshold (10–15 °C) can suppress the ripening process inducing CI and fruit quality losses [10]. Symptoms of avocado CI are skin pitting, blanching, lack of ripening, gray flesh, vascular browning, bad taste, and decay [11] becoming more evident when products are transferred at room temperature.

Other strategies such as employing ethylene inhibitors to delay ripening or using phytohormones to maintain fruit quality have been suggested to alleviate postharvest losses in avocado fruit. Among these strategies is 1-methylcyclopropene (1-MCP), a commercially ethylene inhibitor that acts to delay the ripening process [12]. In this sense, 1-MCP decelerates the respiration rate and weight loss, mitigates physiological discoloration of the mesocarp (gray flesh), and reduces internal CI. Additionally, 1-MCP has shown effectiveness at diminishing fungal infections, enhancing antioxidant balance during fruit storage [13]. It should also be considered that the efficacy of 1-MCP on climacteric fruits is influenced by various factors such as fruit maturity, genotype, compound concentration, application time and method, storage temperature, packaging material, and controlled atmosphere [14].

The synergistic effect of 1-MCP combined with other substances such as melatonin has been studied in zucchini [15] and with methyl jasmonate in pomegranate [16], improving the general 1-MCP effect on the fruit and reducing CI. In avocado, the effect of the combination of 1-MCP with the *Acremonium strictum* Elicitor Subtilisin (AsES) has also been studied, showing higher levels of firmness and reduction in weight loss at the end of storage [17]. Otherwise, different post-harvest treatments based on other elicitors such as γ-aminobutyric acid (GABA) have also been studied. This substance serves as a signaling molecule, playing an important role in plant growth and development, cellular osmoregulation, cellular nitrogen supply, and free radical scavenging under biotic and abiotic stress conditions [18,19]. GABA also has been demonstrated to enhance CI tolerance by stimulating the activities of antioxidant enzymes and maintaining elevated levels of ATP content increasing the energy supply. This mechanism safeguards cell membranes from CI in different species, including peach and orange [20,21]. On the other hand, GABA treatments applied after harvesting have been shown to reduce lipid peroxidation and sustain membrane integrity by stimulating the activity of antioxidant enzymes in different fruits [21,22]. However, as far as we know, there is not any previous study in which the GABA effect on avocado has been studied. For this reason, in this study, we evaluated the effects of post-harvest GABA applications and the impacts of both substances (GABA + 1-MCP) when they were applied as a combined treatment on the shelf life and CI of avocado during cold storage.

## 2. Materials and Methods

### 2.1. Plant Material and Postharvest Treatments

Avocados cv. Hass (*Persea americana* Mill.) were harvested at a commercial plot in Granada (Spain) and transported to the laboratory on the same day of harvest. In the laboratory, the fruit was visually selected considering parameters such as homogenous size and color, with an absence of defects in each piece. After selection (360 fruits), avocados were grouped into 3 replicates of 5 fruits for each treatment and sampling day.

For GABA (Sigma-Aldrich, Madrid, Spain), freshly prepared 1 mM solutions were used for immersion treatments performed for 10 min. This treatment was selected among different GABA concentrations (1–10 mM) and immersion times assayed (10 min and 1 h) in previous experiments (Figures S1 and S2). For the 1-MCP (Smartfresh^TM^, Agrofresh Inc., Philadelphia, PA, USA) and control treatments, distilled water immersions were also applied for 10 min to provide the same conditions. All solutions contained tween-20 (Sigma-Aldrich, Madrid, Spain) 0.05% *v*/*v*. In this sense, 25 avocados were soaked in 10 L of control or GABA solution for each replicate (*n* = 3) and treatment. This ensured thorough coverage and consistent treatment of the fruit. Following treatments, all the fruits were exposed for a drying period at 20 °C (one hour) before being transferred to individual 130 L plastic containers, ensuring consistent conditions across all batches. These containers were then tightly sealed, allowing 1-MCP to take effect over 24 h at 20 °C, while control and GABA 1 mM batches were exposed to ambient air in similarly sealed containers at the same temperature. The application of 1-MCP treatments involved the utilization of commercial tablets, releasing 0.3 µL L^−1^, alongside a commercial activator solution provided by SmartFresh (Agrofresh^®^). For 1-MCP, the optimum concentration applied on these fruits was chosen from those tested on this cultivar by Defilippi et al. [23]. Afterward, these fruits were stored for 35 days at 5 °C and 90% relative humidity, followed by an additional 5-day period at 20 °C for subsequent shelf-life assessments. When stored at 5 °C, CI symptoms could be observed [23], and this temperature was used with simulated shipments studies for this avocado cultivar [24]. All reagents used in this study were of analytical purity.

### 2.2. Postharvest Quality Parameters

The weight loss of individual avocados was expressed as a percentage relative to their initial weight. The results represented the mean ± SE of 15 fruits per replicate (*n* = 3).

Fruit firmness was individually determined using a TX-XT2i texture analyzer (Stable Microsystems, Godalming, UK) equipped with a flat probe with a diameter of 100 mm. The disc descended at a rate of 20 mm min^−1^ until a 5% deformation was achieved. Two readings were taken equidistant along the equatorial region of each fruit. Firmness was expressed as the ratio of applied force to the distance traveled (N mm^−1^).

Respiration and ethylene levels were measured in triplicate by enclosing five avocados per treatment within a 4.6 L plastic container with five fruit per replicate sealed hermetically with a rubber stopper, for a duration of 60 min at room temperature. Following this, a 1 mL gas sample in duplicate (*n* = 3) was injected into a Shimadzu GC-14B gas chromatograph (Shimadzu Europa GmbH, Duisburg, Germany) to determine the CO_2_ concentration, while the other two samples were injected into a Shimadzu GC-2010 gas chromatograph (Shimadzu Europa GmbH, Duisburg, Germany) equipped with an FID detector to measure the ethylene concentration. The chromatographic parameters were detailed by Martínez-Romero et al. [25]. The respiration rate and ethylene production were quantified as mg of CO_2_ kg^−1^ h^−1^ and nL g^−1^ h^−1^, respectively.

The total soluble solids content (TSS) was determined in duplicate in the filtered and centrifuged juice extracted from the mixture of 5 avocado halves for each replicate per batch. The extraction was effectuated after mixing 10 g of avocado pulp with 10 mL of deionized water and finely homogenized (Ultraturrax, T18 basic, IKA, Berlin, Germany). TSSs were measured in filtered and centrifuged juice (10,000 × *g*) at 4 °C for 20 min and using an Atago PR-101 digital refractometer (Atago Co., Ltd. Tokyo, Japan) at 20 °C. TSS results were expressed in g 100 g^−1^. The total acidity (TA) was determined in duplicate through automatic titration (785 DMP Titrino, Metrohm, Herisau, Switzerland) with 0.1 N NaOH until reaching pH 8.1, with 1 mL of juice in 25 mL distilled water. The results were expressed as g 100 g^−1^ of the predominant organic acid (malic acid equivalents) using the following formula: (*M* × 0.067 × *N* × *D*)/*m*) × 100, where *M* is the amount of NaOH used in mL, 0.067 is the acid milliequivalent conversion factor, *N* is the normality of NaOH, *D* is the dilution factor used for the extraction, and *m* is the amount of sample evaluated.

The skin color was assessed using a reflectance Minolta colorimeter (CRC400, Minolta Camera Co., Kantō, Tokyo, Japan) equipped with a D65 illuminant and a CIE 2° standard observer measuring through an 8 mm aperture. Three measurements were taken per avocado, obtaining 15 measures for each replicate (*n* = 3) at three equidistant points along the equatorial area based on CIELab coordinates (CIE L*, CIE a* and CIE b*). CIE hue* (180 + tan^−1^ (b*/a*), if a* < 0), chroma* ((a*^2^ + b*^2^)^1/2^), and ΔE values ((ΔL*)^2^ + (Δa*)^2^ + (Δb*)^2^)^1/2^ were calculated [26], and the results were expressed as the mean ± SE (*n* = 3).

The chlorophyll content was evaluated by extracting a homogeneous mixture of skin samples from 5 avocado halves for each replicate per batch following the method described by Vu et al. [27]. In this sense and after the pigments were extracted by homogenization in methanol for 2 min, samples were centrifuged, and the supernatant was measured in duplicate at the wavelengths of 665.2 and 652.4 in a spectrophotometer (1900 UV/Vis, Shimadzu, Kyoto, Japan). The results were expressed as mg of chlorophyll kg^−1^.

Determination of the susceptibility of avocado to CI was assayed by visual assessment of mesocarp discoloration according to the method described by Hershkovitz et al. [28]. The fruits were sliced lengthwise into two halves to inspect the pulp’s appearance, and the internal damage was evaluated using a 10-point scale as follows: 0 for no damage, 1 for minor damage, 5 for moderate damage, and 10 for severe damage.

Electrolyte leakage (EL) in avocado skin was assessed following the method outlined by Lorente et al. [16] with slight modifications. Fifteen discs were extracted from each replicate of five avocado fruits using a 1 cm diameter cork borer. After three rinses lasting 3 min each with deionized water, the discs were subjected to continuous shaking with 50 mL of deionized water at room temperature. After 60 min, the initial electrical conductivity (C1) was measured using a Crison conductivity meter. Samples were subsequently frozen and then heated to 121 °C for 15 min. Total conductivity (C2) was measured with samples at room temperature (20 °C). EL was calculated as (C1/C2) × 100.

### 2.3. Statistical Analysis

This study was designed with a randomized design. All data presented were expressed as mean ± standard error (SE) (*n* = 3). Analysis of variance (ANOVA) and mean comparisons were conducted using Tukey’s test to identify significant differences (*p* < 0.05). Different lowercase letters denote significant differences between treatments on the same sampling date. All statistical analyses were carried out using the SPSS software package, version 22 (IBM Corp., Armonk, NY, USA).

## 3. Results and Discussion

### 3.1. Effect of 1-MCP and GABA Treatments on Weight Loss and Firmness

The reduction in fruit weight was influenced by two main factors. First, fruit weight loss was connected to vapor-phase diffusion, driven by the vapor pressure difference between the interior and exterior cell fruit compartments. Second, this process was associated with avocado metabolism [29] and the water loss occurring through the stomata, stem scars, and cuticle [3,30]. In this sense, the water loss was determined by the composition and thickness of the cuticle, which was different across different cultivars and maturity stages.

**Figure 1:**
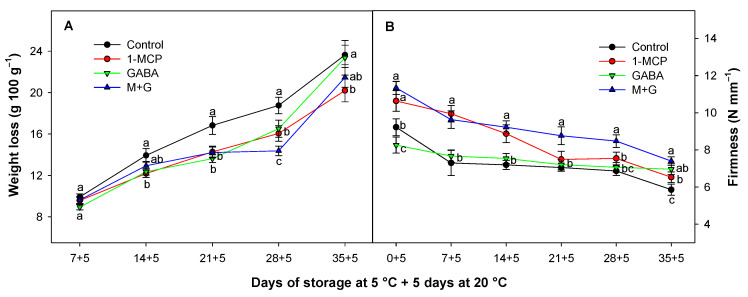
Evolution of weight loss (g 100 g^−1^) (**A**) and fruit firmness (N mm^−1^) (**B**) of Hass avocado treated with distilled water (control), 1-MCP at 0.3 µL L^−1^, GABA at 1 mM, and the combination of 1-MCP and GABA (M+G) during storage at 5 °C plus 5 days at 20 °C. The data are the mean ± SE (*n* = 3). Different lowercase letters show significant differences (*p* < 0.05) among treatments for each sampling date.

Avocado fruit weight loss increased progressively throughout the storage time, showing a linear behavior (Figure 1A). Both 1-MCP and GABA individually delayed weight losses in avocado throughout storage, with significant differences (*p* < 0.05) observed as compared with control fruit. These differences were more pronounced when both treatments (GABA + 1-MCP) were combined after 28 days of cold storage, with 4.38% less weight loss as compared with control fruit (Figure 1A). The delayed weight loss in 1-MCP-treated fruit was linked to a delayed initiation of the respiratory climacteric phase in 1-MCP-treated fruits such as has been observed previously in avocado [28]. In addition to moisture reduction, the metabolism of organic substances like sugars associated to respiration rate played an important role in the decline of fruit weight. Consequently, managing the factors that trigger metabolic processes within the fruit tissue became crucial to controlling weight loss after harvesting [31].

On the other hand, GABA postharvest treatments demonstrated the effect on reducing the degree of membrane lipid peroxidation due to the ability of this compound to improve the antioxidant defense systems, reducing the content of EL and MDA in carambola fruit [32]. In this sense, the higher activity of antioxidant enzymes and the accumulation of bioactive compounds in GABA-treated fruit was related to lower chilling damage, cell membrane permeability, and weight loss in Chinese olive fruit [33].

Texture softening primarily takes place during the fruit ripening process. However, this also makes the fruit more vulnerable to microbial infections and physical damage [31]. In our study, the ripening process of Hass avocados resulted in a gradual reduction in fruit firmness over time, and significant differences in firmness (*p* < 0.05) between the treatments were observed (Figure 1B). Treatments containing 1-MCP maintained significantly higher levels of fruit firmness as compared with the control fruit throughout 14 days of cold storage. Furthermore, when GABA was applied in combination with 1-MCP, fruit firmness was maintained for an additional 7 days. This effect demonstrated synergy between these two substances, as the application of GABA alone did not show any significant difference (*p* > 0.05) as compared with the control batch as it was observed in other fruit species such as peach [22]. While this study did not report any GABA impact on firmness preservation in avocados by itself, previous research indicates that GABA has been linked to maintaining elevated levels of firmness in postharvest mangoes when compared with control samples [34].

Research studies indicate that 1-MCP effectively suppresses the enzymatic activities associated with softening during the ripening process; as a result, the degradation of cell walls is reduced, leading to a delayed softening of fruit [35]. The effect of delayed firmness in avocados treated with 1-MCP has been studied previously [27,36,37], but it has not been studied as a combined treatment with GABA. In this study we observed how the combination of treatments can provide higher values of fruit firmness as compared with control fruit or GABA- and 1-MCP-treated batches when they are applied alone. The GABA pathway is activated, especially against stress responses such as cold storage [38], maintaining cell metabolism against stress conditions in climacteric and non-climacteric fruit [39,40]. For this reason, it seems reasonable to assume that external GABA application could have an improved effect on cell metabolism in MCP-treated tissues, which experience delayed cell wall disassembly compared with control fruit. Consequently, other metabolic parameters, such as respiration and ethylene production, need to be considered to evaluate the impacts of these treatments.

### 3.2. Effect of 1-MCP and GABA Treatments on Respiration Rate and Ethylene

**Figure 2:**
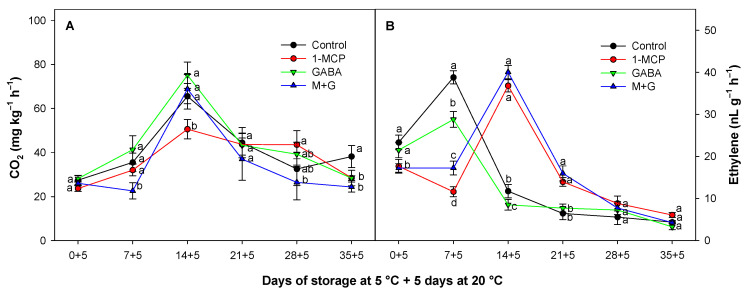
Evolution of respiration rate (mg CO_2_ kg^−1^ h^−1^) (**A**) and ethylene production (nL g^−1^ h^−1^) (**B**) of Hass avocado treated with distilled water (control), 1-MCP at 0.3 µL L^−1^, GABA at 1 mM, and the combination of 1-MCP and GABA (M+G) during storage at 5 °C plus 5 days at 20 °C. The data are the mean ± SE (*n* = 3). Different lowercase letters show significant differences (*p* < 0.05) among treatments for each sampling date.

The climacteric rise of respiration rate was observed on the 14th day of storage at 5 °C plus 5 days at 20 °C in the control and in the treated fruit, resulting in a subsequent decline in both cases. However, 1-MCP significantly (*p* < 0.05) reduced the climacteric rise of the respiration rate as compared with the control batches (Figure 2A). The reduction in the respiratory rate by 1-MCP has been previously studied in avocado [27,41] and in other climacteric fruits such as apricot and kiwi [42,43]. The reduction in the respiration rate can be explained because 1-MCP blocks the ethylene receptors, also decreasing the physiological processes in fruits and vegetables [44]. While GABA treatments did not show significant differences (*p* > 0.05) when applied alone as compared with control fruit, when combined with 1-MCP, they demonstrated the most effective (*p* < 0.05) reduction in the overall respiratory production of the experiment. The combined treatment (GABA + 1-MCP) peaked higher than in fruit treated only with 1-MCP, showing an influenced pattern similar to GABA-treated fruit at that moment. The application of 1-MCP has been demonstrated to increase GABA content in both apples and pears [45,46]. Additionally, exogenous applications of GABA stimulate the GABA pathway, thereby promoting the tricarboxylic acid (TCA) cycle in GABA-treated plants. This stimulation contributes to an increase in the cellular energy status, which demands immediate substrates to obtain energy especially under cold storage [47,48]. For this reason, it is reasonable to hypothesize that with the combined treatment, both technologies together provided additional GABA content stimulated endogenously, with 1-MCP and with GABA exogenous applications reducing the cell stress. Under cold-related stress, maintaining ATP content is crucial in various plant species [49]. This is achieved by regulating the mitochondrial oxidative defense system and preserving the mitochondrial structure. A deficiency in ATP has been shown to increase the content of reactive oxygen species (ROS) [50]. Hence, these findings collectively highlight the intricate interplay between 1-MCP, GABA, and ATP in plants, for enhancing plant resilience under suboptimal temperatures. The respiration rate of climacteric fruits is influenced by ethylene production. In climacteric fruits such as avocado, there is an increase in ethylene production at the onset of ripening [7]. On the other hand, the application of exogenous ethylene increased the respiratory rate, whereas the use of 1-MCP decreased it [51,52].

In this study, ethylene production exhibited typical ethylene climacteric patterns, and the peaks occurred at 7 days of storage in control and GABA treatments and after 14 days of storage in 1-MCP-treated batches alone and in combination with GABA immersions (Figure 2B). The same delay was observed in peak ethylene production by Jeong et al. [53], Hershkovitz et al. [27], and Zhang et al. [36] where the climacteric peak of ethylene occurred 6, 4, and 7 days later, respectively, in the avocado fruit treated with 1-MCP as compared with the control batch. Although 1-MCP delayed the ethylene peak and production, it did not quantitatively reduce the ethylene peak in 1-MCP-treated batches, as reported in previous studies [27,51,53].

When GABA treatments were applied alone, they significantly (*p* < 0.05) reduced ethylene production at the beginning of the study. However, after 14 days of storage, there were no significant differences (*p* > 0.05) as compared with control fruit. In other studies, GABA postharvest treatments were also effective in reducing ethylene production in apples [54] and guavas [55]. This effect has been observed in tomatoes treated with GABA, leading to a lower accumulation of key metabolic intermediates such as 1-aminocyclopropane-1-carboxylic acid (ACC), which slightly reduces ethylene production in this fruit. On the other hand, 1-MCP, as a potent inhibitor of ethylene action, reduces ethylene activity, thereby slowing down fruit ripening, affecting also metabolic substrates [44].

### 3.3. Effect of 1-MCP and GABA Treatments on Total Soluble Solids (TSSs) and Total Acidity (TA)

**Figure 3:**
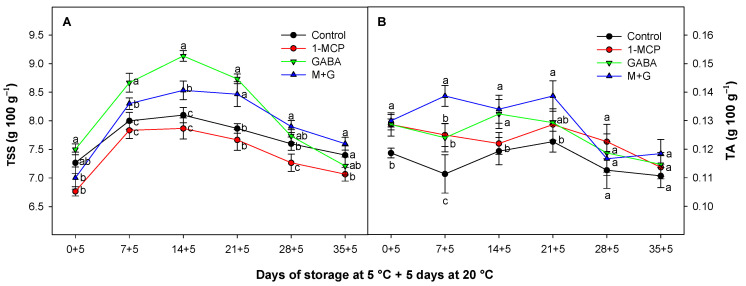
Evolution of total soluble solids (TSSs) (g 100 g^−1^) (**A**) and total acidity (TA) (g 100 g^−1^) (**B**) of Hass avocados treated with distilled water (control), 1-MCP at 0.3 µL L^−1^, GABA at 1 mM, and the combination of 1-MCP and GABA (M+G) during storage at 5 °C plus 5 days at 20 °C. The data are the mean ± SE (*n* = 3). Different lowercase letters show significant differences (*p* < 0.05) among treatments for each sampling date.

In the early stages of storage, a gradual increase in TSSs was observed (Figure 3A). As the avocado ripened, this process intensified, and TSSs reached their peak. However, as the avocado continued its ripening process, some sugars may have converted into other compounds or been lost, potentially resulting in a decline in TSSs toward the end of the storage period. Similar values and fluctuations were also previously observed in Hass avocados [56,57], which seemed to occur specifically in late season avocado sucrose content with or without 1-MCP treatments [58]. In this sense, the TSS value of GABA-treated fruits showed a consistent increase with higher values than those observed in both control and 1-MCP-treated fruits from day 0 to day 14, followed by a decline toward the end of the storage period. The 1-MCP-treated fruit alone displayed the lowest TSS level along the storage. The increased TSS levels in GABA-treated batches could be attributed to GABA as an immediate metabolic substrate, potentially resulting in a reduced respiration rate delaying the breakdown of sugars and leading to TSS accumulation, as observed in other fruit species such as peach, apple, and persimmon [20,54,59]. This association was linked to a stimulation of the GABA-shunt pathway covering energy demands reducing stress and metabolism [48]. In this sense, we previously observed in this study a delayed ethylene pattern in GABA-treated avocado batches, as well as in the respiration process in GABA + 1-MCP batches (Figure 2A,B).

On the other hand, the TA values of the control fruit slightly decreased along the storage (Figure 3B) in consonance with other authors [57]. These values (0.10–0.14%) were similar than those observed by Salameh et al. [56] in Hass avocados grown in different locations (0.09–0.17%). However, GABA-treated fruit and MCP-treated batches exhibited significantly higher TA values as compared with the control fruit. Furthermore, the GABA + 1-MCP batch consistently displayed the highest TA levels, as compared with the other evaluated batches in general, confirming the delayed metabolism in GABA and 1-MCP treatments alone or combined. In fact, the additional positive effect observed when these substances were combined could be attributed to the synergistic benefits observed when applied individually. In this sense, GABA treatments also delayed TA evolution in other fruit species as persimmon fruit during storage [59] and after 1-MCP treatments [44] including avocado [60], as we have observed in this study, specifically for the GABA + 1-MCP-treated batch.

### 3.4. Effect of 1-MCP and GABA Treatments on Skin Color and Chlorophyll Content

**Figure 4:**
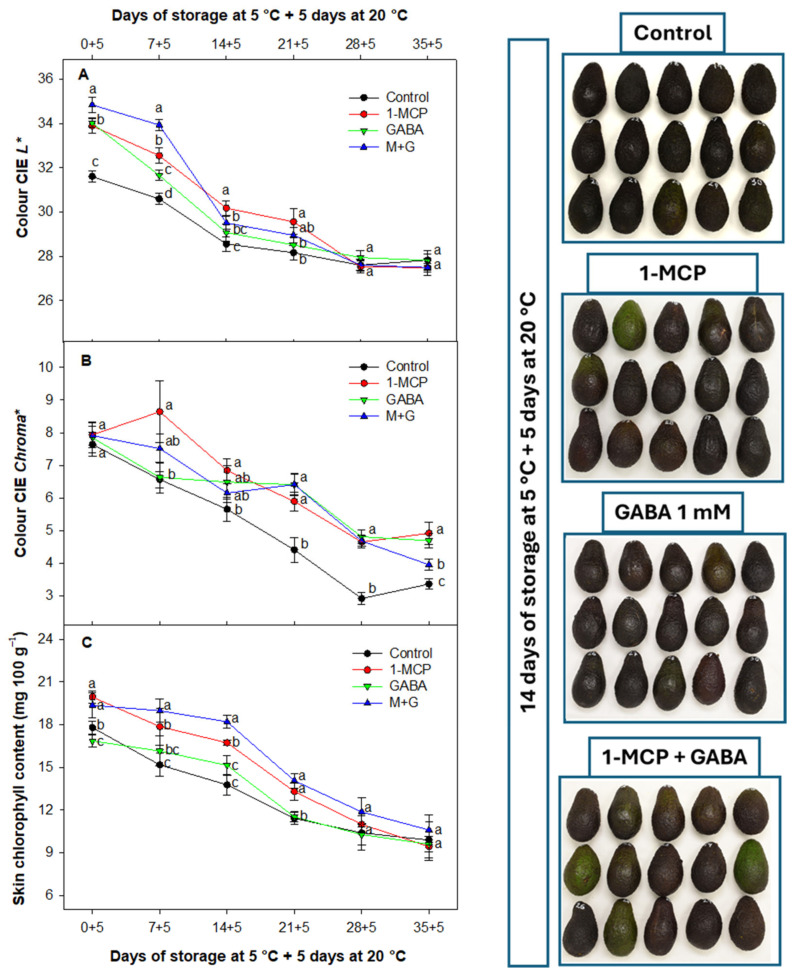
Appearance and evolution of skin external color CIE L* (**A**), CIE Chroma* (**B**), and skin chlorophyll content (mg 100 g^−1^) (**C**) of Hass avocado treated with distilled water (control), 1-MCP at 0.3 µL L^−1^, GABA at 1 mM, and the combination of 1-MCP and GABA (M+G) during storage at 5 °C plus 5 days at 20 °C. The data are the mean ± SE (*n* = 3). Different lowercase letters show significant differences (*p* < 0.05) among treatments for each sampling date.

The change in skin color of Hass avocados during ripening, transitioning from green to purple and ultimately black, occurs due to a reduction in chlorophyll concentration initially, succeeded by a rise in the presence of the anthocyanin known as cyanidin 3-O-glucoside [61]. Regarding exocarp color, the decline in color CIE L* represented the loss of lightness in avocado during storage [61]. In this study, color CIE L* maintained lower values during storage for the control fruit as compared with the fruit treated with GABA or 1-MCP both individually and applied as a combination of treatments (Figure 4A). On the other hand, CIE chroma* values were also delayed in all GABA- and 1-MCP-treated batches (Figure 4B). In this sense, 1-MCP treatments alone or combined with GABA displayed higher medium values than control fruit. External color was affected by GABA and 1-MCP in a similar way; batches containing 1-MCP alone or combined with GABA showed significant (*p* < 0.05) higher values as compared with GABA when it was applied alone, especially after short-term storage. This delayed pattern can be observed also regarding other color parameters evaluated as CIE hue* and ΔE* (Figure S3).

Accordingly, decreases in the total chlorophyll content of avocado skin were observed during storage (Figure 4C). However, this decrease was significantly delayed (*p* < 0.05) in fruit treated with the combination of 1-MCP and GABA as compared with fruit batches treated individually with these compounds and control fruit, mainly after short-term storage. The 1-MCP showed a stronger effect in delaying the reduction in chlorophylls as compared with fruit batches treated only with GABA, although both treatments controlled this parameter with higher medium values in general than those observed for control fruit. Toward the end of the study, no differences were observed between all the different fruit batches.

For many fruits, the reduction in chlorophyll and the evolution of colored pigments are crucial components of the ripening process. The slower progression of CIE L* evolution has been associated with a reduced rate of fruit weight loss and a delayed ripening process [62]. The 1-MCP inhibits the loss of greenness, or yellowing, in most plant products. Consequently, effective 1-MCP applications delay all these processes but without causing them to become permanently inhibited [44]. The 1-MCP has been observed to delay external color in Hass avocado in different studies through diminished production of ethylene [44,60]. Hershkovitz et al. [27] also elucidated that the delayed color in avocado fruit treated with 1-MCP was related to a delayed ethylene production and a lower chlorophyllase activity.

On the other hand, although the effect of GABA treatments has not been evaluated previously in avocados, several studies have recently confirmed the effectiveness of 40 mM GABA in delaying color evolution and chlorophyll degradation as a preharvest treatment in apples [63] and as a postharvest treatment in pistachios with 10 mM GABA [64]. These concentrations were considerably higher than those applied in this postharvest study (1 mM). The additional effectiveness observed with the combined treatment (1-MCP + GABA) has not been previously studied in any fruit species, but it could be attributed to the synergistic interaction of both technologies producing a combined positive effect.

### 3.5. Effect of 1-MCP and GABA Treatments on Electrolyte Leakage and Chilling Injury

During ripening, senescence, and in response to stress, a sequence of oxidative metabolic reactions occurs, impacting the integrity of cell membranes. This leads to changes in membrane permeability and results in cellular dysfunctions, including the unregulated movement of electrolytes [65]. On the other hand, one of the frequently observed phenomena associated with CI is an elevation in membrane permeability, characterized by an increase in the release of ions.

EL has been traditionally considered a qualitative indicator of CI in avocados and is a major marker of cell membrane deterioration under cold stress [28]. This parameter in avocado fruit has been shown to be associated with membrane permeability, indicating a strong correlation between EL with fruit softening and ethylene production [66]. For this reason, ethylene blocking substances such as 1-MCP have been previously reported as a successful technology for reducing CI in avocado [27,51].

**Figure 5:**
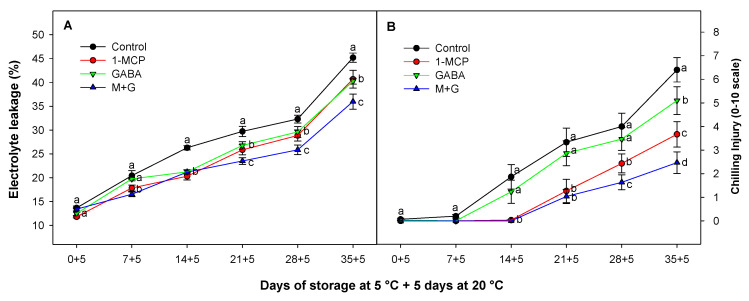
Evolution of electrolyte leakage (%) (**A**) and CI (0–10 scale) (**B**) of Hass avocado treated with distilled water (control), 1-MCP at 0.3 µL L^−1^, GABA at 1 mM, and the combination of 1-MCP and GABA (M+G) during storage at 5 °C plus 5 days at 20 °C. The data are the mean ± SE (*n* = 3). Different lowercase letters show significant differences (*p* < 0.05) among treatments for each sampling date.

In this study, we observed a comparable trend between CI symptoms in avocado fruit, manifested as mesocarp discoloration, and increased EL values. Control fruit exhibited higher levels of EL than treated fruit along 35 days of storage since this parameter was reduced by 10.02 and 10.96% for 1-MCP and GABA treatments, respectively, when applied alone (Figure 5A). However, when these two substances were combined, the reduction in CI reached 20.52%. Although the effects of GABA and 1-MCP were similar in reducing EL, the 1-MCP treatment was significantly (*p* < 0.05) more effective in fruit treated with 1-MCP than GABA when CI was evaluated (Figure 5B). In this sense, after 35 days of cold storage, CI was reduced by 42.81% and 20.31% for 1-MCP and GABA treatments, respectively, when applied alone. Additionally, a synergistic effect was observed in this parameter when combining these treatments, reducing CI by 61.56%.

Loss of membrane integrity facilitates enzyme–substrate interaction, triggering flesh darkening and discolorations because of enzymatic browning [67]. This phenomenon is stimulated by increased production of ROS during postharvest storage and reduced activity of antioxidant enzymes in avocado [67] and other plant species [30,68]. For this reason, the status of the antioxidant and energy balance plays a critical role in protecting cellular membranes and enhancing tolerance to chilling conditions [48]. In this sense, GABA can induce the expression of antioxidant enzymes or have indirect antioxidant effects by modulating metabolic pathways related to oxidative stress [48]. The potential of GABA treatment has been studied as an effective tool to positively protect cell membranes from lipid peroxidation in blood orange and loquat fruit [69,70]. This effect has been related to an improvement of the antioxidant balance, increasing the activity of the antioxidant enzymes in fruit treated with GABA at preharvest [71] or postharvest [69,72]. This maintenance of antioxidant balance combined with an increase in the GABA-shunt pathway in GABA-treated fruit has been shown to provide extra energy in cells to address cold stress [48].

The 1-MCP has been studied in avocados and has been observed to mitigate membrane damage in avocado fruit through the reduction in EL, reducing, in turn, the prevalence of physiological mesocarp discoloration (gray flesh) and internal CI [27,28]. These results were coincident with those obtained in this study, where it could be observed that treatments with 1-MCP alone or in combination with GABA significantly (*p* < 0.05) reduced avocado CI during storage (Figure 5B).

**Figure 6:**
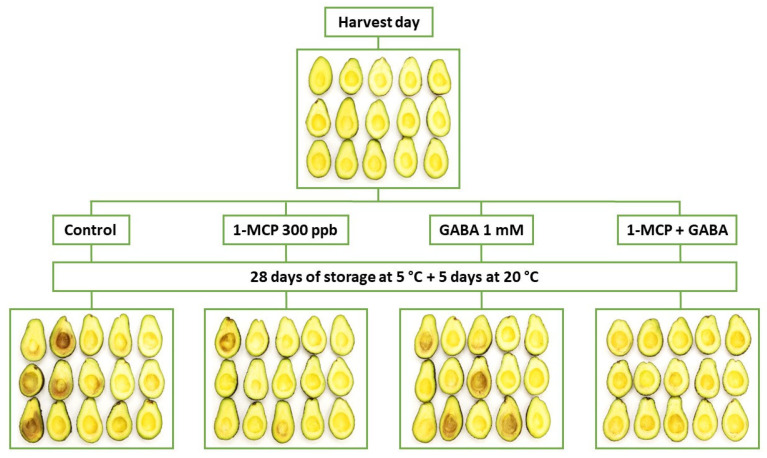
Internal visual aspect of Hass avocado at harvest day and avocado treated with distilled water (control), 1-MCP at 0.3 µL L^−1^, GABA at 1 mM, and the combination of 1-MCP and GABA (M+G) after 28 days of storage after 5 °C plus 5 days at 20 °C.

These results can be visually verified in Figure 6, where the effectiveness of the different treatments assayed on CI was also observed. GABA plays an important role in reducing CI symptoms through better maintenance of cell membrane integrity in plant products such as peach [20], banana [73], and zucchini [74]. For these reasons, the increased effect combining both technologies may be related to the additive benefits observed with the combined treatment.

## 4. Conclusions

In conclusion, the results of this study suggested the significant potential of combined applications of 1-methylcyclopropene and γ-aminobutyric acid in enhancing the overall quality and extending the shelf life of Hass avocados. The results indicated that the combined application of 1-methylcyclopropene and γ-aminobutyric acid impacted with synergistic effects, resulting in improved preservation of fruit weight, firmness, and respiratory rate regulation. Additionally, the treatments influenced biochemical parameters contributing to the maintenance of fruit quality attributes and delaying external color changes, enhancing visual appeal and marketability. Notably, the reduction in electrolyte leakage and chilling injury highlights the role of these treatments in enhancing membrane integrity and minimizing physiological disorders during storage. Overall, the results suggested that the combined application of 1-methylcyclopropene and γ-aminobutyric acid could be an effective postharvest management strategy for Hass avocados, offering growers and stakeholders a viable approach to optimize fruit quality and extend market availability.

## Data Availability

The original contributions presented in the study are included in the article/Supplementary Material, further inquiries can be directed to the corresponding author.

## Supplementary Materials

The following supporting information can be downloaded at https://www.mdpi.com/article/10.3390/foods13162485/s1: Figure S1: Preliminary experiment. Weight loss (%) evolution of ‘Hass’ avocado; Figure S2: Preliminary experiment. Chilling injury symptoms (0–10 scale) of ‘Hass’ avocado; Figure S3: Evolution of skin external colour CIE hue* (A) and ΔE (B) of ‘Hass’ avocado.
