# Supplementary material for: Innovative Postharvest Management for Hass Avocado at the Preclimacteric Stage: A Combined Technology with GABA and 1-MCP
Source: Foods. 2024 Aug 7;13(16):2485. doi: 10.3390/foods13162485 (PMC11354002; doi:10.3390/foods13162485)
Supplement: Supplementary file 1 [file foods-13-02485-s001.zip › foods-3125188-supplementary.pdf]

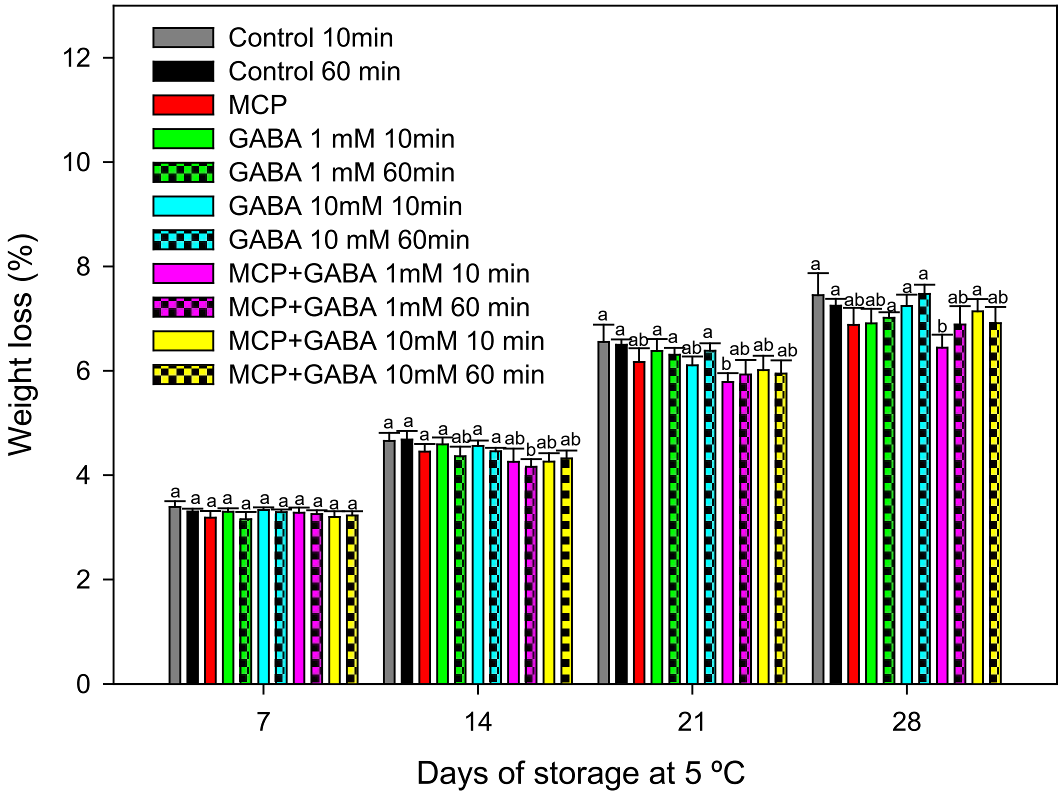

Figure S1. Preliminary experiment. Weight loss (%) evolution of 'Hass' avocado treated for 10 or 60 minutes with distilled water (control and 1-MCP), GABA (1 and 10 mM), and fruit treated with 1-MCP (0.3 μL L^{-1}) alone or combined with GABA treatments during refrigerated storage at 5 ºC. Data are the mean ± SE (n = 3). Different lowercase letters show significant differences ($p < 0.05$) among treatments.

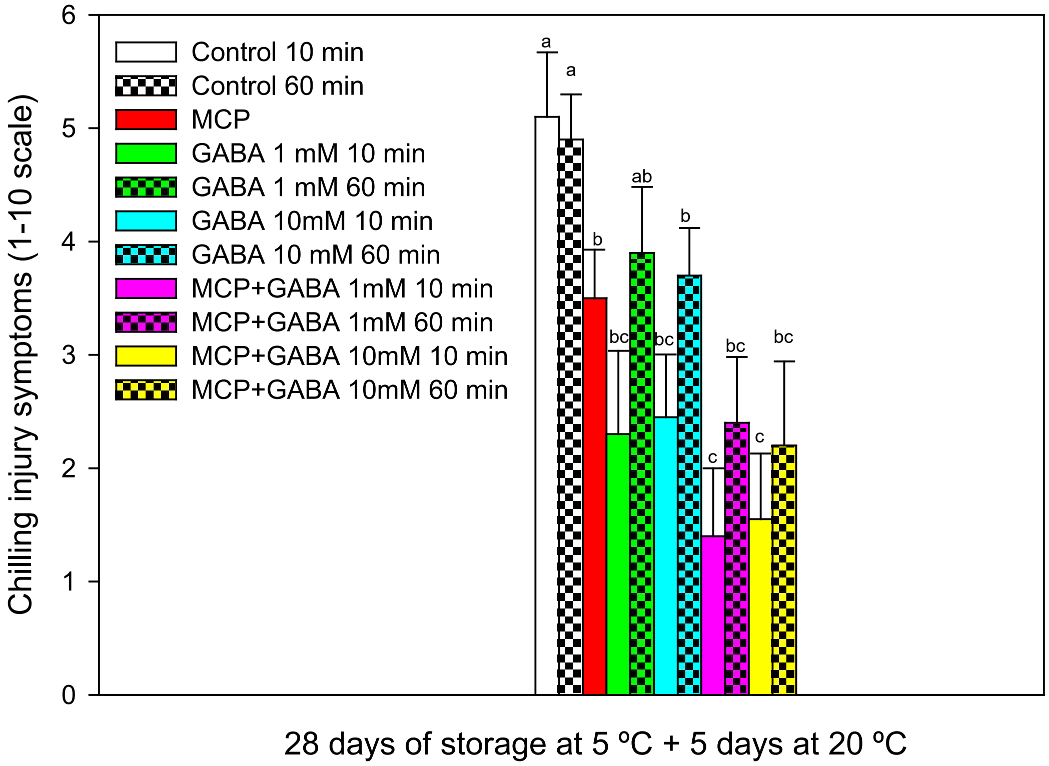

Figure S2. Preliminary experiment. Chilling injury symptoms (0-10 scale) of 'Hass' avocado treated for 10 or 60 minutes with distilled water (control and 1-MCP), GABA (1 and 10 mM), and fruit treated with 1-MCP (0.3 μL L^{-1}) alone or combined with GABA treatments after 28 days of storage at 5 °C plus 5 days at 20 °C. Data are the mean ± SE (n = 3). Different lowercase letters show significant differences (p < 0.05) among treatments.

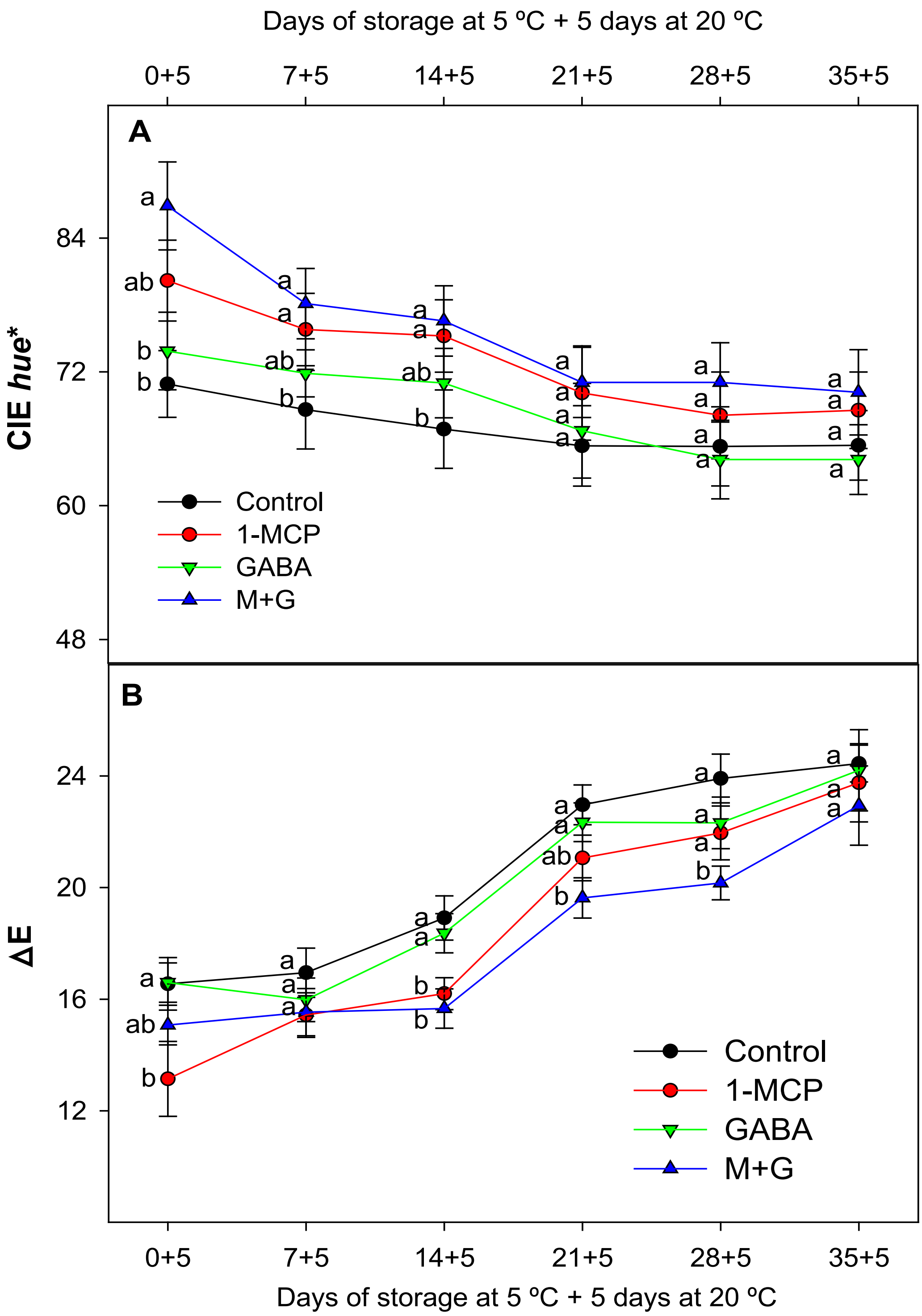

Figure S3. Evolution of skin external colour CIE *hue** (A) and ΔE (B) of 'Hass' avocado treated with distilled water (Control), 1-MCP at 0.3 μL L–1, GABA at 1 mM and the combination of 1-MCP and GABA (M+G) during storage at 5 °C plus 5 days at 20 ° C. Data are the mean ± SE (n = 3). Different lowercase letters show significant differences ($p < 0.05$) among treatments for each sampling date.
